# Membrane-embedded TSPO: an NMR view

**DOI:** 10.1007/s00249-020-01487-0

**Published:** 2020-12-22

**Authors:** Gwladys Rivière, Garima Jaipuria, Loren B. Andreas, Andrei Leonov, Karin Giller, Stefan Becker, Markus Zweckstetter

**Affiliations:** 1 Senior Research Group of Translational Structural Biology in Dementia, Deutsches Zentrum für Neurodegenerative Erkrankungen (DZNE), Von-Siebold-Str. 3a, 37075 Göttingen, Germany; 2 Department of Neurology, University Medical Center Göttingen, University of Göttingen, Waldweg 33, 37073 Göttingen, Germany; 3 Department of NMR-Based Structural Biology, Max Planck Institute for Biophysical Chemistry, Am Fassberg 11, 37077 Göttingen, Germany

**Keywords:** Cholesterol, Dynamics, Solid-state NMR, Structure, Lipid, Membrane protein, Neurodegeneration, TSPO

## Abstract

Translocator Protein (18 kDa) (TSPO) is a mitochondrial transmembrane protein commonly used as a biomarker for neuroinflammation and is also a potential therapeutic target in neurodegenerative diseases. Despite intensive research efforts, the function of TSPO is still largely enigmatic. Deciphering TSPO structure in the native lipid environment is essential to gain insight into its cellular activities and to design improved diagnostic and therapeutic ligands. Here, we discuss the influence of lipid composition on the structure of mammalian TSPO embedded into lipid bilayers on the basis of solid-state NMR experiments. We further highlight that cholesterol can influence both the tertiary and quaternary TSPO structure and also influence TSPO localization in mitochondria-associated endoplasmic reticulum membranes.

## Introduction

Translocator Protein (18 kDa) (TSPO), initially identified and named as a peripheral benzodiazepine receptor, is a transmembrane protein mainly located in the outer mitochondrial membrane (Anholt et al. 1986; McEnery et al. 1992). The protein has been renamed as 18-kDa translocator protein (TSPO) to better reflect its cellular activities and tissue distribution (Papadopoulos 2006). TSPO is present in most peripheral organs (e.g., adrenal glands, lungs and heart) and is also expressed in microglial cells in the healthy brain, but is especially abundant in steroidogenic tissues (Banati 2002). In several neurological diseases, TSPO is markedly upregulated in microglia and astrocytes (Gui et al. 2020; Martín 2010; Wilms 2003). As a result, TSPO became one of the most used biomarkers for imaging of neuroinflammation using positron emission tomography (PET) (Wilms 2003; Schain and Kreisl 2017).

Over the years, a large number of TSPO PET radioligands have been synthesized to improve our knowledge regarding the role of neuroinflammation in central nervous system diseases and to assess the efficacy of novel anti-inflammatory therapeutic strategies. A common drawback of all TSPO radioligands is their sensitivity to a single-nucleotide polymorphism (rs6971) that results in an amino-acid substitution on the target protein: A147T-TSPO (Guo 2013; Owen 2012; Kreisl 2013). This polymorphism generates three genotypes: homozygous high-affinity binders (HABs), heterozygous mixed-affinity binders (MABs), and homozygous low-affinity binders (LABs) (Owen 2010,2011). LABs are rare and make up 9% of Caucasians, 6% of African Americans and 0.001% Han Chinese and Japanese^9^. Excluding LABs is important in clinical studies because brain uptake of the radioligands is too low to be quantified. Recently, two third-generation TSPO radioligands: [^11^C]-ER176 and [^18^F]-GE180 showed improved detection of TSPO signals in LABs. [^11^C]-ER176, a quinazoline analog of PK11195, was identified to bind TSPO across all rs6971 genotypes in membranes prepared from human brain tissue (Fujita 2017; Ikawa 2017; Boutin 2015). This radioligand has an improved lipophilicity (LogD decreased from 3.97 to 3.55) and increased the accuracy of quantification (Fujita 2017). [^18^F]-GE180, a tricyclic indole compound, has exhibited higher signal-to-noise ratios than [^11^C]PK11195 in preclinical models of stroke and Alzheimer’s diseases (Boutin 2015; Chaney 2019; Sridharan 2017; Liu 2015; López-Picón 2018). In healthy human controls, [^18^F]GE-180 has low brain penetration (Sridharan 2019; Zanotti-Fregonara 2018; Feeney 2016; Fan 2016); whereas, a markedly increased uptake of [^18^F]GE-180 was observed in lesions of patients, which have HAB, MAB and LAB genotype, with multiple sclerosis (Unterrainer 2018a; Vomacka 2017) or glioma (Albert 2017; Unterrainer 2018b, 2019). The validity of [^18^F]GE-180 for TSPO imaging is debated, as the origin of this increased signal is uncertain (Albert 2019; Zanotti-Fregonara 2019).

Despite its extensive use as a target in imaging studies, TSPO function is not well understood. TSPO was long considered to play an important role in the translocation of cholesterol through the mitochondrial membrane and thus execute a critical step in steroidogenesis (Costa et al. 2018; Fan et al. 2015). However, the direct role of TSPO in neurosteroidogenesis has been challenged by the development of TSPO knockout mouse models (Costa et al. 2018; Papadopoulos et al. 2018; Banati 2014). At the same time, TSPO genetic deletion and PET imaging studies in various disease models highlighted that TSPO is involved in cellular bioenergetics, associated mitochondrial functions such as redox homeostasis and apoptosis, and also plays a role in innate immune processes of microglia (Repalli 2015; Mukhin et al. 1989; Betlazar et al. 2020; Gut et al. 2015).

TSPO ligands have also been shown to have neuroprotective properties in various animal models of neurodegeneration (Jung 2020; Arbo et al. 2015). Ro5-4864 was found to attenuate the accumulation of amyloid-beta plaques and decrease microglial activation in a mouse model for Alzheimer’s disease. This was accompanied by improved behavior and cognition (Barron 2013). Administration of Emapunil (XBD173), on the other hand, ameliorated microgliosis and neuroinflammation in the MPTP mouse model for Parkinson’s disease, and protected against neuronal loss in the substantia nigra, dopamine depletion, and motor deficits (Gong 2019). Treatment with XBD173 (or Etifoxine) also improved clinical and neuropathological features in rodent models of multiple sclerosis through inhibition of inflammation, elevation of neurosteroids and prevention of demyelination (Bader 2019; Daugherty 2013; Leva 2017), and prevented microglial reactivity after injury in a mouse model of retinal degeneration (Scholz 2015; Akhtar-Schäfer et al. 2018). The preclinical studies indicate that TSPO is a potential target to delay neurodegenerative disease by the regulation of neuroinflammation, apoptosis, and steroidogenesis (Repalli 2015; Arbo et al. 2015; Bader 2019).

Decrypting the structure of TSPO in lipid bilayers is essential to understand its biological role and to design ligands for diagnostic and therapeutic applications. So far, the structure of TSPO from mouse (*m*TSPO) and from bacterial homologues—all solubilized in detergent—has been resolved by NMR spectroscopy and X-Ray crystallography (Jaremko et al. 2014,2015a,b; Guo 2015; Li et al. 2015; Xia 2019). Here, we describe solid-state NMR studies of *m*TSPO embedded into lipid bilayers, to gain insights into the lipid- and ligand-dependent structure of mammalian TSPO in a near-native environment.

## Materials and methods

### NMR sample preparation

Expression and purification of ^13^C/^15^N-labeled *m*TSPO were performed as described previously (Jaremko et al. 2014; Lacapère 2001). To obtain liposomes with homogeneous lipid composition and distribution, we dissolved the lipids in a glass vial in chloroform/methanol = 1/1 (v/v). The organic solvent was removed by a constant nitrogen stream followed by lyophilization. The resulting lipid film was dissolved in TSPO buffer by repeated sonication in a water bath. The resulting liposomes (prepared with either 1,2-dinervonoyl-sn-glycero-3-phosphocholine; 24:1 (*cis*) PC 850,399 Avanti) or sarcolemma lipid mix (34.7% 18:1 PC, 16.6% 18:1 PE, 2.3% 18:0 PI, 2.3% 18:1 PS, 1% 18:1 PIP2, 9.1% 18:0 SM, 31.9% Chol), percentage in molar ratio) were incubated with the foscholine-12-solubilized protein at a protein/lipid molar ratio of 1:20 for two hours at room temperature. After removal of the detergent with biobeads (BioRad), liposomes were pelleted by centrifugation at 125.000×*g*. The liposome pellet was washed with 10 mM sodium phosphate pH 6.0, pelleted and transferred into an NMR rotor.

## Solid-state NMR spectroscopy

NMR samples contained ~ 12–15 mg of protein packed in a 3.2 mm rotor with DSS for temperature calibration. Solid-state NMR experiments were recorded either on a 850 MHz wide-bore spectrometer equipped with 3.2 mm triple-resonance probe (Bruker Biospin) or on a 950 MHz Bruker Avance III HD standard-bore spectrometer equipped with a 3.2 mm (^1^H, ^13^C, ^15^ N) E_free_ triple-resonance probe (Bruker Biospin). The (2D) ^13^C–^1^H dipolar-assisted rotational resonance experiments (Takegoshi et al. 2001,2003) were recorded at 5 °C and 35 °C with a mixing time of 20 ms. The following 90° pulse widths were used: 2.5 μs for ^1^H, and 4.5 μs for ^13^C. ^1^H decoupling strengths were 80–100 kHz. Spectra were processed in Topspin (Bruker) and analyzed with CcpNmr-Analysis (Stevens 2011).

## Results and discussion

### Interaction of cholesterol with mTSPO

**Fig. 1:**
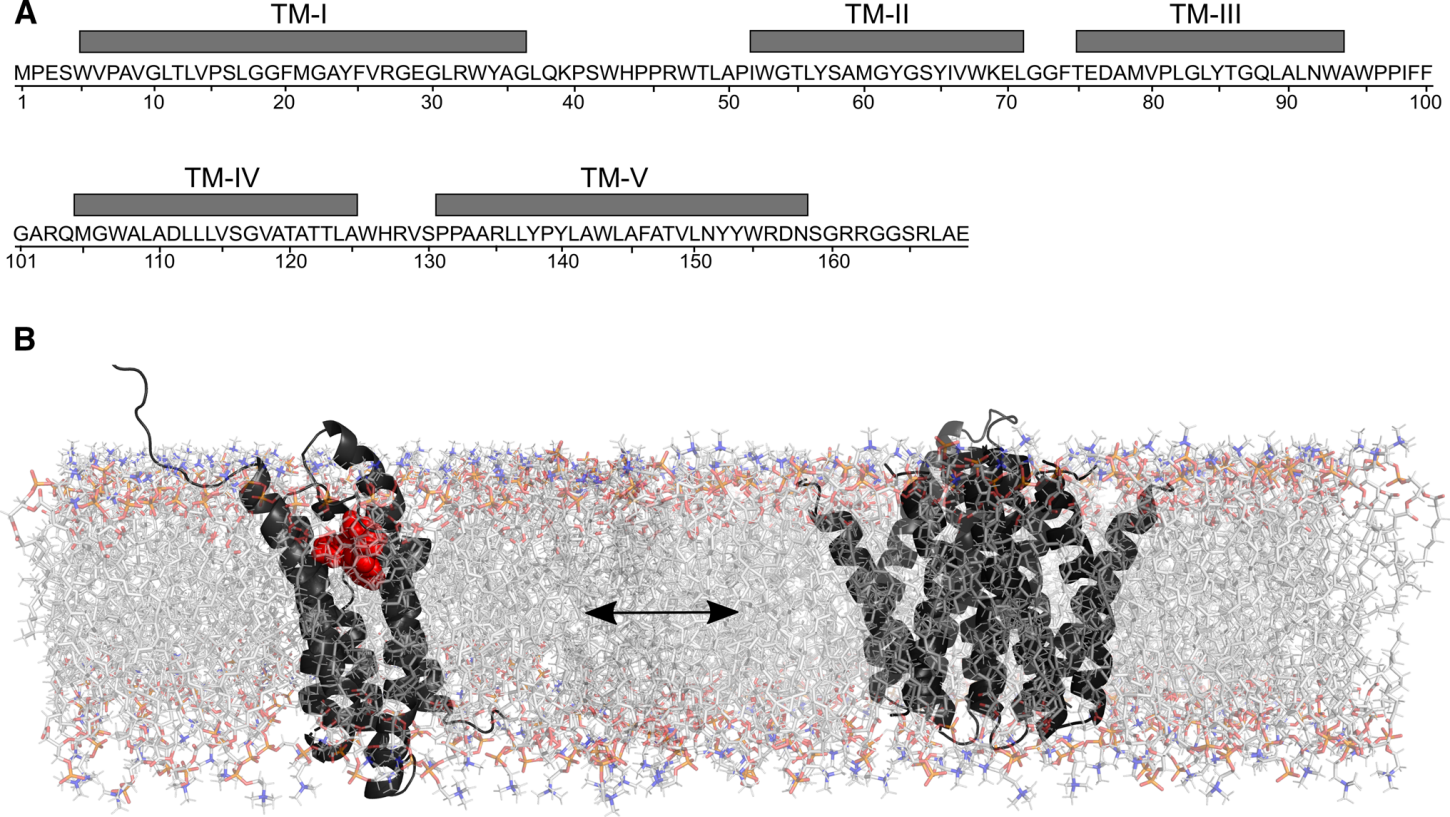
The structure of membrane-embedded TSPO. *m*TSPO amino acid sequence highlighting the location of its five transmembrane helices (**a**). Structural model of the *m*TSPO monomer–dimer equilibrium in DMPC bilayer membrane (**b**). DMPC lipids represented in stick, PK11195 in red spheres and *m*TSPO displayed in black cartoon representation

The structure, dynamics and function of membrane proteins are influenced by their lipid environment (Palsdottir and Hunte 2004). Hence, to gain insight into the activity of TSPO and its interaction with ligands it is crucial to study its conformation in the near-native environment of lipid bilayers. Solid-state NMR in combination with computational analysis suggested that *mouse* TSPO (mTSPO) reconstituted into liposomes and in dodecyl-phosphocholine detergent micelles fold into a bundle of five transmembrane helices (Jaremko et al. 2015a) (Fig. 1a). In these NMR studies, *m*TSPO is structurally stabilized through binding of high-affinity radioligands: PK11195 (Jaremko et al. 2015a,^2016^; Murail, et al. 1778) or DA1106 (Jaipuria 2017). While *m*TSPO is monomeric in detergent systems (Jaremko et al. 2015a,^2016^), a fraction of *m*TSPO associates into oligomeric species in lipids (Jaipuria 2017; Teboul 2012). High-resolution solid-state NMR further revealed that *m*TSPO bound to DA1106 exists in a dynamic monomer/dimer equilibrium in DMPC liposomes (Fig. 1b). The monomer/dimer equilibrium is concentration dependent, is mediated by the ^83^GxxxG^87^ motif in the transmembrane helix TM-III, and inhibited in the G87V mutant of *m*TSPO (Jaipuria 2017). Cholesterol, known to bind mammalian TSPO with nanomolar affinity (Lacapère 2001), influences *m*TSPO oligomerization by shifting the equilibrium towards the monomeric form (Jaipuria 2017). In addition, binding of cholesterol to the CRAC (cholesterol recognition amino acid consensus) motif of *m*TSPO (Jamin 2005) induces distal structural changes on helix TM-III, that might be a consequence of the transition from the dimeric to the monomeric form (Jaipuria 2017; Jaipuria et al. 2018a). Although details of these structural changes are unknown, they might involve changes in TM helix orientation, especially helices TM-II and TM-V (Jaipuria 2017). Interestingly, cholesterol binding is not restricted to the CRAC motif, as demonstrated using paramagnetic cholesterol analogs (Jaipuria et al. 2018a): paramagnetic broadening in the spectra of wild type and G87V-mTSPO suggested that cholesterol binds to an additional site in monomeric *m*TSPO (Jaipuria et al. 2018a). This site is located on helix TM-III and, thus, not available in dimeric *m*TSPO, where TM-III is buried in the dimer interface. Thereby, depending on the local protein and cholesterol concentration, the GxxxG motif of mammalian TSPO will be available to interact with cholesterol or other outer mitochondrial membrane proteins and, thus, can influence TSPO activity.

### Solid-state NMR spectroscopy of mTSPO in different lipid environments

*m*TSPO reconstituted in either detergent or 1,2-dimyristoyl-sn-glycero-3-phosphocholine (DMPC) liposomes displays broad NMR lines when no high-affinity ligand is bound (Xia 2019; Murail et al. 1778; Jaipuria 2017; Jaremko et al. 2015c). The broad NMR lines suggest the presence of internal dynamics and/or exchange between multiple conformations that is intermediate on the NMR time scale. The structural flexibility might arise from a hydrophobic mismatch between *m*TSPO and the DMPC lipid bilayer. To minimize hydrophobic mismatch, adaptations in the membrane protein can occur including tilting of TM helices or/and rotation of side chains at the ends of TM helices (Lee 2003).

To increase the membrane thickness and, thus, potentially stabilize TSPO’s TM helices, we reconstituted *m*TSPO into 1,2-dinervonoyl-sn-glycero-3-phosphocholine (24:1 PC). With a chain length of 24 carbons, 24:1 PC achieves a hydrocarbon region thickness of 37.5 ± 1 Å and a molecular surface area of 67.7 ± 1.9 Å^2^ (Lewis and Engelman 1983). We also reconstituted *m*TSPO in a sarcolemma lipid mixture (34.7% 18:1 PC, 16.6% 18:1 PE, 2.3% 18:0 PI, 2.3% 18:1 PS, 1% 18:1 PIP2, 9.1% 18:0 SM, 31.9% Chol), to investigate the influence of anionic lipids on the *m*TSPO structure. ^13^C–^13^C correlation spectra of *m*TSPO were recorded at 35 °C and 5 °C and compared to previous results (Jaipuria 2017), in which *m*TSPO had been reconstituted into DMPC:cholesterol (20:10 molar ratio).

**Fig. 2:**
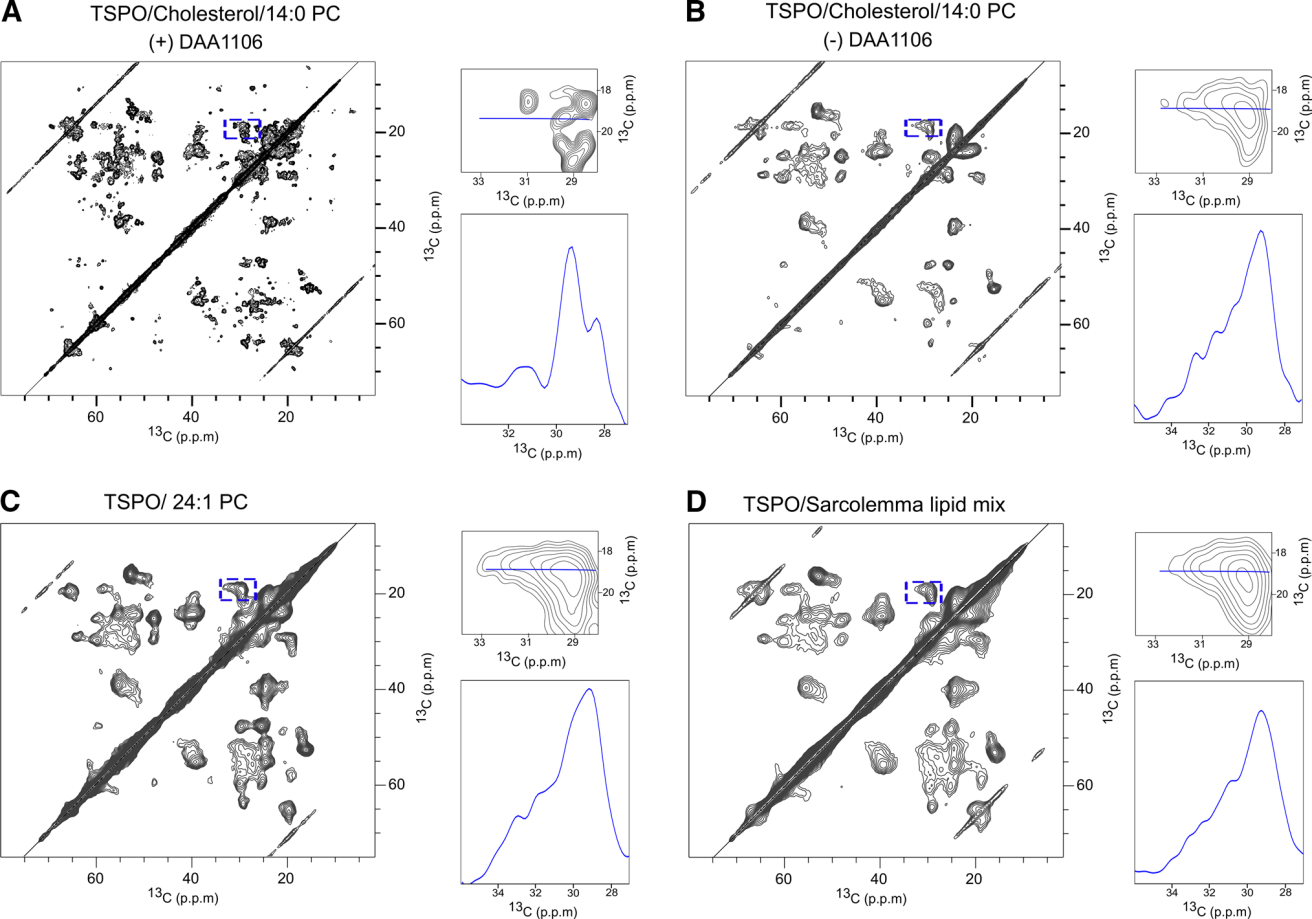
^13^C–^13^C correlation spectra of membrane-embedded *m*TSPO at 5 °C. **a**, **b** ^13^C–^13^C PDSD spectrum of *m*TSPO reconstituted into DMPC-cholesterol liposomes in the presence (**a**) or absence (**b**) of *N*‐ (2,5‐dimethoxybenzyl)‐*N*‐ (5‐fluoro‐2‐phenoxyphenyl)acetamide (DAA1106); protein:DMPC:cholesterol molar ratios of 1:20:10. Spectra were recorded with a spinning speed of 11 kHz (Jaipuria 2017). **c**, **d** ^13^C–^13^C DARR spectra of *m*TSPO reconstituted into 24:1 PC (**c**), and into a sarcolemma lipid mixture (**d**). To visualize the NMR linewidth, peaks framed in blue were enlarged and a 1D horizontal projection of these is displayed

^13^C–^13^C correlation spectra recorded at 5 °C for *m*TSPO bound to DA1106 displayed a large number of defined NMR signals (Fig. 2a). In contrast, the same spectra recorded for *m*TSPO reconstituted into 24:1 PC (Fig. 2c), or sarcolemma lipid mixture (Fig. 2d), displayed mostly unresolved cross peaks, suggesting the presence of multiple *m*TSPO conformations. Further comparison with the spectra of *m*TSPO in DMPC:cholesterol (Fig. 2b), showed that the use of 24:1 PC (Fig. 2c) slightly narrowed the NMR lines, pointing to a partial stabilization of the *m*TSPO structure in 24:1 PC.

**Fig. 3:**
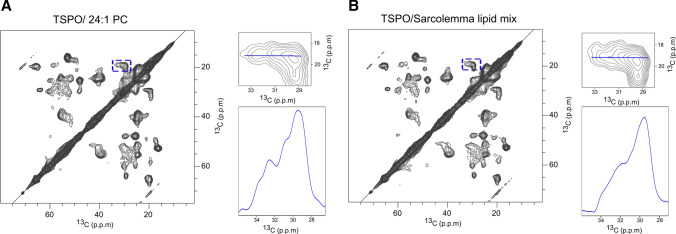
^13^C–^13^C DARR correlation spectra of *m*TSPO reconstituted into 24:1 PC (**a**), and into a sarcolemma lipid mixture (**b**), at 35 °C. To visualize the NMR linewidth, peaks framed in blue were enlarged and a 1D horizontal projection of these is displayed

Next, we recorded the ^13^C–^13^C correlation spectra at 35 °C (Fig. 3), i.e., above the phase transition of 24:1 PC, which occurs at ~ 24 °C (Lewis and Engelman 1983). While the NMR cross peaks are still broad when compared to those of *m*TSPO in complex with DAA1106 (Fig. 2a), the resolution slightly improved when compared to the spectra at 5 °C (Fig. 3a when compared to Fig. 2c). Similar to the spectra recorded at 5 °C, the ^13^C–^13^C correlation spectrum of *m*TSPO in the sarcolemma lipid mixture (Fig. 3b) was of lower quality when compared to *m*TSPO in 24:1: PC (Fig. 3a). One possible reason for the lower spectral quality in the sarcolemma lipid mixture is that the anionic lipids modulate the conformational exchange in *m*TSPO.

Taken together, the analysis reinforces our previous observations that *m*TSPO in complex with a high affinity ligand is most suitable for high-resolution NMR studies. In addition, the variation in the quality of the NMR spectra of *m*TSPO in different lipid bilayers stresses the potential influence of the lipid environment on the structure and, thus, activity of mammalian TSPO.

### Lipid composition influences mTSPO structure and dynamics

The current results together with previous data show that the lipid composition influences the structure and dynamics of membrane-embedded *m*TSPO. *m*TSPO reconstituted into DMPC liposomes exchanges between a monomeric and dimeric structure. This equilibrium depends on both the protein and cholesterol concentration (Jaipuria 2017). Cholesterol binds to *m*TSPO, promotes dissociation of the *m*TSPO dimer and causes structural changes (Jaipuria et al. 2018a). Notably, cholesterol regulates the formation of mitochondria-associated endoplasmic reticulum membranes (MAMs), which are sites of close proximity between the endoplasmic reticulum and mitochondria (Fujimoto et al. 2012; Aufschnaiter 2017; Area-Gomez and Schon 2016). Bilayers deficient in these raft-like microdomains are linked to neurodegenerative diseases by contributing to mitochondrial dysfunction and subsequent neuronal decay (Aufschnaiter 2017; Area-Gomez and Schon 2016; Szymański 2017). MAMs incorporate a distinct set of proteins including among others VDAC, inositol 1,4,5-trisphosphate receptor, grp75, Mfn2 (Aufschnaiter 2017; Stoica 2014) and TSPO (D’Eletto 2018). VDAC and TSPO have been shown to interact directly with each other to regulate both mitochondrial structure and function (Gatliff 2015; Shoshan-Barmatz et al. 2019).

The conformational heterogeneity of *m*TSPO is also present when the protein is reconstituted—in the absence of a high-affinity ligand—into 24:1 PC or into a sarcolemma lipid mixture (Figs. 2, 3). Increasing the membrane thickness using 24:1 PC lipids slightly decreased the conformational heterogeneity when compared to DMPC or the sarcolemma lipid mixture (Fig. 3). The lipid composition of sarcolemma shows features in common with MAMs, such as high content in PC, cholesterol and sphingolipid. Nevertheless, the lower quality of the spectra of *m*TSPO in the sarcolemma lipid mixture when compared to *m*TSPO reconstituted into DMPC/cholesterol liposomes suggests that the *m*TSPO structure is less stable in the sarcolemma lipid mixture. This surprising result might be explained by the presence of anionic lipids such as PE, PI, PS, PIP2. Further studies of *m*TSPO embedded into MAM lipids will be needed to understand both direct and indirect (e.g., changes in membrane fluidity and thickness) effects of lipids on the structure and dynamics of mammalian TSPO.

## Conclusions

Cholesterol is an important regulator of TSPO structure as well as the TSPO and VDAC co-localization in mitochondria-associated membranes. Solid-state NMR provided unique insights into the tertiary and quaternary structure of membrane-embedded *m*TSPO and its interaction with cholesterol (Jaipuria 2017; Jaipuria et al. 2018a,b). Future studies are required in order to fully characterize the interaction of cholesterol with mammalian TSPO and its impact on the orientation and dynamics of TSPO’s transmembrane helices. In addition, atomic-scale insight into the VDAC/TSPO complex embedded into MAM lipids is urgently required. We believe that solid-state NMR will play a critical role in these studies, because of its unique ability to investigate protein structures in native-like membranes and to probe both rigid and flexible protein structures. Moreover, the recent advancements in solid-state NMR instrumentation and radiofrequency pulse sequences have significantly enhanced the sensitivity and resolution of solid-state NMR experiments (Mandala et al. 2018), empowering the study of large membrane-embedded protein complexes.

## Abbreviations

NMR: Nuclear magnetic resonance
TSPO: Translocator protein—18 kDa
*m*TSPO: Mouse TSPO
CRAC: Cholesterol recognition amino acid consensus
DAA1106: *N*‐ (2,5‐Dimethoxybenzyl)‐*N*‐ (5‐fluoro‐2‐phenoxyphenyl)acetamide
PET: Positron emission tomography
HAB: High-affinity binders
MAB: Heterozygous mixed-affinity binders
LAB: Low-affinity binders
DMPC: 1,2-Dimyristoyl-sn-glycero-3-phosphocholine
TM: Transmembrane
PC: Phosphatidyl-choline
PE: Phosphatidyl-ethanolamine
PI: Phosphatidyl-inositol
PS: Phosphatidyl-serine
PIP2: Phosphatidylinositol 4,5-bisphosphate
SM: Sphingomyelin
VDAC: Voltage-dependent anion channel
MAM: Mitochondria-associated endoplasmic reticulum membranes
